# How research-based theatre is a solution for community engagement and advocacy at regional medical campuses: The Health and Equity through Advocacy, Research, and Theatre (HEART) program

**Published:** 2018-03-27

**Authors:** Allison Brown, Natalie Ramsay, Michael Milo, Mo Moore, Rahat Hossain

**Affiliations:** 1 McMaster University – Michael G. DeGroote School of Medicine, Niagara Regional Campus, Ontario, Canada; 2 Department of Health Research Methods, Evidence, and Impact, McMaster University, Ontario, Canada

## Abstract

**Background:**

Regional medical campuses are often located in geographic regions that have different populations than the main campus, and are well-positioned to advocate for the health needs of their local community to promote social accountability within the medical school.

**Methods:**

At the Niagara Regional Campus of McMaster University, medical students developed a framework which combined research, advocacy, and theatre to advocate for the needs of the local population of the regional campus to which they were assigned. This involved a qualitative study using semi-structured interviews with homeless individuals to explore their experience accessing the healthcare system and using a transformative framework to identify barriers to receiving quality healthcare services. Findings from the qualitative study informed a play script that presented the experiences of homeless individuals in the local health system, which was presented to health sciences learners and practicing health professionals. Participants completed two instruments to examine the utility of this framework.

**Results:**

Research-based theatre was a useful intervention to educate current and future health professionals about the challenges faced by homeless individuals in the region. Participants from both shows felt the framework of research-based theatre was an effective strategy to promote change and advocate for marginalized populations.

**Conclusion:**

Research-based theatre is an innovative approach which can be utilized to promote social accountability at regional medical campuses, advocating for the health needs of the communities in which they are located, with the added bonus of educating current and future health professionals.

## Introduction

Medical schools across the globe have “the obligation to direct their education, research, and service activities towards addressing the priority health concerns of the community, region, and/or nation they have a mandate to serve.”^1^,^2^ Nationally, social accountability is a requirement of all Canadian medical schools as defined in the Standards and Elements for accreditation by the Committee on the Accreditation of Canadian Medical Schools.^3^ Additionally, the Health Advocate CanMEDS role encourages physicians to use their expertise and influence to advance the health and well-being of individual patients, communities, and populations.^4^ Core fundamentals of this skill include responding to the health needs of their community and identifying the determinants of health of their patients. Despite this, medical schools continue to be challenged in finding ways to incorporate social accountability and advocacy into their curriculum and measure the outcomes of those changes on training physicians as health advocates.^5^

Through distributed medical education (DME), future physicians can be trained in smaller communities beyond the main campus to promote future careers in these smaller centres. To address physician shortages in North America, there has been a rapid growth of distributed campuses over the last 10 years. This permits all or part of the medical degree to be completed outside of the main medical campus.^6^ Across Canada, there are currently 16 regional medical campuses affiliated with 12 of the 17 Canadian medical schools.^7^ The placement of distributed medical campuses in the community seeks to establish partnerships with both the local community, businesses, and government. These partnerships can help to establish trust and encourage collaboration on health policy and system development for these communities.^8^ One study from the University of Toronto found that medical students who were trained in a rural or distributed setting were 20% more likely to discuss experiences as a health advocate when reflecting on their rotations.^9^ Fostering the engagement of students in health advocacy is an important aspect of ensuring social accountability in its trainees. Regional campuses are well-positioned to partner with the local community in which they are situated to identify areas which the community feels are important to them. This fosters collaboration with community partners to respond to and advocate for their healthcare needs. Distributed campus trainees are afforded more opportunities for community involvement and advocacy through experiential learning than their main campus counterparts.^9^

The Niagara Regional Campus of the Michael G. DeGroote School of Medicine at McMaster University is located in the Niagara region in St. Catharines, Ontario, approximately one hour from the main teaching campus in Hamilton, Ontario. Medical students begin their training for the three-year medical degree at the Niagara Regional Campus following their first of five pre-clinical units at the main campus, in November of their first year. The remainder of the pre-clerkship curriculum and core clerkship rotations are then completed at the regional campus, meaning that students spend approximately 30 months in the Niagara region. During their time as medical students, many become involved in extracurricular activities, including research and volunteer work. Although many of the extracurricular opportunities for medical students are available at the main campus in Hamilton, Niagara students have the opportunity *to* lead their own innovations locally.

The Niagara region is a large geographic area composed of three main urban cities and several rural areas. The three hospitals of the Niagara Health System in St. Catharines, Welland, and Niagara Falls serve over 400,000 patients annually. While the Niagara region can be characterized by its beautiful landscape, vineyards, and famous waterfalls, it is also home to high rates of poverty and housing issues.^10^ The *Living in Niagara Report* is a community research publication focusing on development goals and community issues every three years.^10^ It has consistently demonstrated homelessness as a major social issue affecting the entire region. Over half of the homeless population has major chronic health conditions and encounter daily challenges when accessing healthcare such as drug and alcohol abuse, lack of housing, and food insecurity.^11^ Healthcare in the homeless population is consistently demonstrated to have many barriers to access, as many feel as if their views about their health are being disregarded and believe that their healthcare providers lack compassion for their situations.^8,12^ Given the disparities identified in the *Living in Niagara Report* and the literature surrounding increased morbidity and mortality for homeless individuals, healthcare for the homeless was identified by a team of medical students from the Niagara Regional Campus as a priority area for advocacy in the Niagara region.

The purpose of this paper is to describe how medical students combined research-based theatre with advocacy at a regional campus, including the impact of the intervention on current and future health professionals, and discuss ways in which this framework can be utilized by other medical schools to advocate for the local needs of the patient populations that they serve.

## Methods

**Figure 1:**
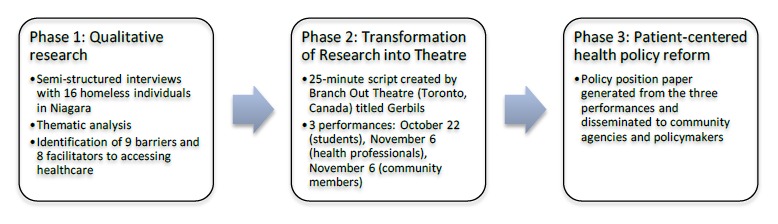
HEART framework

Health and Equity through Advocacy, Research, and Theatre (HEART) is a student-led initiative which seeks to advocate for marginalized populations through research-based theatre (www.heart-program.com). The process of HEART is as follows (Figure 1):

1. Phase I - Use qualitative, semi-structured interviews to understand the experiences of homeless individuals in the healthcare system across the Niagara Region and transform findings into research-based theatre. Research-based theatre uses formal theatrical production to mount the live performance of research and the researcher’s interpretations of data for an audience.^13^ The script is composed of qualitative data (e.g., interview transcripts, field notes) that have been analyzed and dramatized. The characters in the production are generally representative of research participants, but portrayed by actors. Research-based theatre has been used successfully to promote best practice in clinical education and knowledge translation in qualitative health research.^14^,^15^
2. Phase II - Present the play (“Gerbils”) to three audiences: Future health professionals (students), current health professionals (practicing clinicians), and individuals from the homeless community. Audience members replace an actor and improvise during scenes of the play which illustrate barriers or challenges in accessing healthcare. Policies are generated by a “policy panel” (composed of community, social services, government, and healthcare representatives) as they observe the interventions acted out by audience members. These policies are subsequently voted upon by the audience, and those that are “passed” by a majority vote are drafted into a policy position paper representing the needs of the community.
3. Phase III - Patient-centered health policy reform: The policy position paper can be provided to community members, agencies, and policymakers to act and advocate on behalf of the issues that were identified by the community and the solutions that they generated.

### Ethical approval

Ethical approval was granted by the Hamilton Integrated Research Ethics Board for Phases I and II of the program (HIREB File #1267). All study participants consented to their participation in the research.

### Data collection and analysis

During Phase I of the program, 16 individuals who struggled with housing participated in face-to-face, semi-structured interviews which lasted 30-60 minutes in duration. Purposeful and criterion sampling strategies were used to identify participants for the interviews. Eligibility criteria included individuals over the age of 18 who self-identified as homeless or vulnerably housed, and had an interaction with the healthcare system while homeless or vulnerably housed between 2011 and 2016. Eligible participants were recruited through three shelters across the Niagara region prior to being approached for consent. Interviews were transcribed and analyzed inductively to identify barriers and facilitators to accessing healthcare in the Niagara region.^16^ A transformative framework was used as the guiding paradigm, recognizing that experience and construction of knowledge is influenced by power and that the objective of the research was to advocate for this marginalized population and promote change through political debate and discussion.^17^ Nine barriers and eight facilitators were identified through thematic analysis of the interview transcripts; narrative analysis was then used to transfer these research findings into a theatre script.^18^ The transcripts and qualitative findings were given to Branch Out Theatre (Toronto, Ontario) who created a 25-minute script, titled *Gerbils*.

To evaluate the influence of the intervention on current and future health professionals (Show #1 and Show #2), two instruments were administered following the performance to collect information about the intervention as a means for engagement and advocacy. The Legislative Theatre Questionnaire (LTQ) surveys participants about the impact of the experience by asking five questions with Likert scale response options, ranging from Strongly Disagree (1) to Strongly Agree (5).^14^ The Public and Patient Engagement Evaluation Tool (PPEET©) was licensed under a Creative Commons Attribution.^19^ The PPEET includes 14 questions to assess the activity as a means for engagement, with the reverse response options on a 5-point Likert scale, ranging from Strongly Agree (1) to Strongly Disagree (5). Data from the LTQ and PPEET were entered into IBM SPSS 24. Item descriptive statistics using means and standard deviations were calculated for both instruments.

## Results

### Research-based theatre for health professional learners and practicing clinicians

Three separate performances of *Gerbils* were held during Fall 2016 for three audiences. Audience members from the first and second show were invited to enjoy the play, and participate in a research study evaluating the impact of research-based theatre (Table 1).

**Table 1:** Participant demographics

Age	Show 1 (n=39)	Show 2 (n=21)
18-24	69%	0%
25-29	10%	5%
30-39	8%	5%
40-49	0%	10%
50-59	0%	19%
60+	0%	14%
Did not specify	13%	48%

**Gender**		

Female	64%	67%
Male	36%	33%
Unspecified	0%	0%

**Discipline**		

Nursing	28%	10%
Medicine	69%	57%
Social Work	0%	14%
Unspecified	3%	0%
Other	0%	19%

Audience members who attended the first performance were largely medical and nursing students, whereas members who attended the second performance, for health professionals, included a few allied health professions.

Results from the LTQ demonstrate that participants from both shows agree that they gained insight into what it is like to struggle with housing, and that the performance influenced how they would interact with homeless individuals and other marginalized populations in a clinical setting (Table 2). Participants also agreed that research-based theatre was an effective strategy for educating health professionals about providing care for the homeless, and a strategy which can promote policy change.

**Table 2:** Results from the Legislative Theatre Questionnaire

		Means (SD)	
		Show 1 (n=39)	Show 2 (n=21)
1.	I have gained insight about what it is like to be a patient who struggles with housing.	4.05 (0.61)	4.06 (0.87)
2.	I feel that the knowledge I gained from this play will impact the way I interact with homeless and other marginalized patients.	4.10 (0.89)	4.00 (0.77)
3.	I think that using research-based theatre is an effective way of educating people about caring for a person who is homeless (in comparison to traditional methods such as through literature).	3.97 (0.97)	4.39 (0.98)
4.	The *quality* (as opposed to content) of this production positively affected my learning.	4.06 (0.67)	4.19 (0.83)
5.	Legislative theatre would be an effective way to promote policy change.	3.42 (1.02)	4.13 (1.03)

Show 1 = healthcare students, Show 2 = healthcare professionals

1: Strongly Disagree; 2: Disagree; 3: Neither Agree nor Disagree; 4: Agree; 5: Strongly Agree

Results from the PPEET suggest that the experience was well received, especially by the practicing health professionals (Table 3). Confidence in how participant input would be used was low in the student group, however both groups did feel this activity would make a difference and was a good use of their time.

**Table 3:** Results from the Public and Patient Engagement Evaluation Tool (PPEET)

		Means (SD)	
		Show 1 (n=39)	Show 2 (n=21)
1.	The purpose of the activity was clearly explained	2.28 (0.91)	1.59 (0.51)
2.	The supports I needed to participate were available (e.g. travel, child care, etc.)	2.34 (1.16)	2.40 (0.91)
3.	I had enough information to contribute to the topic being discussed	2.20 (0.86)	1.81 (0.54)
4.	I was able to express my views freely	2.36 (0.96)	1.63 (0.50)
5.	I feel that my views were heard	2.44 (0.88)	1.56 (0.51)
6.	A wide range of views on the topic were expressed	2.75 (1.18)	1.94 (1.03)
7.	I feel that the input provided through this activity will be considered by the organizers	1.89 (0.75)	1.75 (0.45)
8.	The activity achieved its stated objectives	2.39 (0.80)	1.75 (0.45)
9.	I understand how the input from this activity will be used	3.03 (1.13)	1.94 (0.57)
10.	I think this activity will make a difference	2.54 (0.85)	2.00 (0.63)
11.	As a result of my participation in this activity, I am better informed about barriers and facilitators to accessing healthcare for homeless individuals.	2.14 (0.91)	2.00 (0.89)
12.	As a result of my participation in this activity, I have greater trust in my ability to influence health policy change	2.97(1.01)	2.38 (0.89)
13.	Overall, I was satisfied with this activity	2.20 (0.83)	1.81 (0.54)
14.	This activity was a good use of my time	2.24 (0.92)	1.63 (0.50)

1: Strongly Agree; 2: Agree; 3: Neither Agree nor Disagree; 4: Disagree; 5: Strongly Disagree

After the policy panel generated draft policies during the performance and presented them to participants, a total of eight policies were endorsed between the three shows. The policy position paper has since been published and distributed to community organizations, and medical students continue to advocate for these recommended policies to be adopted throughout the Niagara region.^20^

## Discussion

Distributed campuses should advocate for the needs of the communities in which they are located. The use of research-based, legislative theatre was effective in educating current and future health professionals about the difficulties faced by those who struggle with housing, live in poverty, and have challenges navigating the healthcare system. Both healthcare students and healthcare professionals found the HEART framework of research-based theatre to be effective in conveying empathy, healthcare disparities, and promoting change in policies which directly affect the social climate of a community. Both audiences felt that this experience will influence how they interact with homeless individuals in a clinical setting. While results from the second show seem to show a stronger impact, this may be due to process issues experienced during the first show, such as time constraints, which may have influenced participant’s perceptions.

For healthcare providers to be advocates, they need to better understand the challenges encountered by their patients. Dharamsi et al. discuss that a focus of medical education should be a particular attention paid towards addressing inequalities in healthcare and marginalized populations, or those impacted by the social determinants of health.^5^ Medical students and healthcare providers found the research-based play to be educational in improving their understanding of the experiences of homeless patients. The HEART framework may be useful in medical education for training future physicians on how to be advocates for their patients who may have difficulty or be unable to advocate for themselves.

The HEART framework is a potential approach for engaging medical students in social accountability and advocacy by combining research and community engagement. Students and professionals alike were found to develop insight into health disparities of Niagara’s homeless population, as well as develop empathy through research-disseminated theatre. Distributed medical education was in part created to address the disparity of health resources in more rural and remote communities, where patients face even more barriers and challenges in accessing equitable healthcare.^6^ Community engagement is difficult to incorporate into medical education; however, its integration may lead to better retention of physicians in the community in which they were trained. Based on the preliminary results, the HEART framework is an effective method for community engagement and social accountability with medical students and healthcare professionals. As such, medical schools could use this framework as a unique opportunity to better meet the objectives of distributed undergraduate and continuing medical education.

As regional campuses are intimately intertwined with their community, their responsibility to be socially accountable and educate their students regarding community determinants of health is paramount. Furthermore, as students identify these community needs through their own experiential learning, they may be more engaged in working to ameliorate these gaps in care. Although the educational resources developed by the HEART program are effective in educating all healthcare students and professionals, the ease of its implementation could be hindered in a traditional academic centre, where medical education tends to focus on a specialist and hospital-oriented model rather than a generalist and community-oriented one.^21^ Regional campuses can use the HEART framework to advocate for the unique needs of their home communities; the approach is an effective method to get students involved in advocacy work, promote social accountability at local levels, as well as promoting the synthesis of healthcare and the humanities.

### Limitations

Findings from Phase I of this study are limited due to our sampling strategy, as we were only able to interview individuals recruited through shelters, and thus could not capture homeless individuals who do not use these services. The majority of these services are also located in the mid-north end of the Niagara region, and thus our sample was restricted to these areas and did not represent the lived experiences of individuals in the South of Niagara, where experience in the healthcare system may be different. We also did not explore the differences between subgroups of homeless participants, including race or gender. Further studies with more participants that span the entirety of the region would allow for more generalizability of the findings. Furthermore, subgroup analyses were not conducted on the PPEET data due to the small sample size obtained. Future iterations of HEART and research-based theatre interventions may be more adequately powered to allow for these comparisons. Data for shows 1 and 2 were collected immediately post-intervention, and may not be valid when extrapolated for consideration of long-term impact on health professionals.

### Conclusion

Overall, HEART was a successful strategy to engage with the community, give voice to a marginalized population - homeless individuals - in the Niagara region, educate current and future health professionals about the lived experiences of patients in our own healthcare system, and advocate for change at a local level. A research-based theatre approach may be utilized by other distributed sites of medical schools to foster community engagement, advocacy for the community’s needs, and promote policy change.

Regional campuses may be better positioned to advocate for the health needs of their community and promote social accountability. Research-based theatre is an innovative approach which may be easily utilized by students at distributed sites to give a voice to the health needs of the community and promote patient-centered policy reform with the added benefit of educating current and future health professionals about their experience in the healthcare system, promoting empathy, understanding, and better healthcare.

## References

[1] Boelen C, Heck J Defining and measuring the social accountability of medical schools. Geneva, Switzerland; 1995.

[2] Cappon P, Parboosingh J, McMurrat R, et al Social Accountability: A Vision for Canadian Medical Schools. Ottawa, ON; 2001.

[3] Committee on Accreditation of Canadian Medical Schools CACMS Standards and Elements: Standards for Accreditation of Medical Education Programs Leading to the M.D. Degree. 2015.

[4] Sherbino J, Bonnycastle D, Côte B, Flynn L, Hunter A, Ince-Cushman D, et al The CanMEDS 2015 Health Advocate Expert Working Group Report. 2014;

[5] Dharamsi S, Ho A, Spadafora SM, Woollard R The Physician as Health Advocate: Translating the Quest for Social Responsibility Into Medical Education and Practice. Acad Med. 2011;86(9):1108–13. 21785306 10.1097/ACM.0b013e318226b43b

[6] Cheifetz CE, McOwen KS, Gagne P, Wong JL Regional Medical Campuses: A New Classification System. Acad Med. 2014;89(8):1140–3. 24826857 10.1097/ACM.0000000000000295

[7] Group on Regional Medical Campuses (GRMC) Official List of Regional Medical Campuses [Internet]. 2013 Available at: https://www.aamc.org/members/grmc/ [Accessed May 30, 2017].

[8] Acosta O, Toro P Letʼs Ask the Homeless People Themselves: A Needs Assessment Based on a Probability Sample of Adults. Am J Community Psychol. 2000;28(3). 10.1023/A:1005105421548 10945121

[9] Girard-Pearlman Banack J, Albert M, Byrne N, Walters C Canadian Medical Education Journal A Conceptual Model for Teaching Social Responsibility and Health Advocacy: An Ambulatory/Community Experience (ACE). Can Med Educ J. 2011;2(22):e53-e64.

[10] Niagara Connects Living in Niagara Report: Critical Indicators for Reflecting on Life in Niagara. Niagara, Ontario; 2014.

[11] Hwang SW, Martin RE, Tolomiczenko GS, Hulchanski JD The Relationship Between Housing Conditions and Health Status of Rooming House Residents in Toronto on JSTOR. Can J Public Heal. 2003;94(6):436-440. 10.1007/BF03405081 PMC6979903 14700243

[12] Nickasch B, Marnocha SK Healthcare experiences of the homeless. J Am Acad Nurse Pract. 2009;21(1):39–46. 19125894 10.1111/j.1745-7599.2008.00371.x

[13] Saldaña J Dramatizing Data: A Primer. Qualitative Inquiry. 2003;9(2): 218-36. doi:10.1177/1077800402250932

[14] Colantonio A., Kontos P. C., Gilbert J. E., Rossiter K., Gray J., & Keightley M. L After the Crash: Research-Based Theater for Knowledge Transfer. Journal of Continuing Education in the Health Professions. 2008;28(3):180-85. doi:10.1002/chp.177 18712795

[15] Kontos P. C., & Naglie G Expressions of Personhood in Alzheimers Disease: An Evaluation of Research-Based Theatre as a Pedagogical Tool. Qualitative Health Research. 2007;17(6):799-811. doi:10.1177/1049732307302838 17582022

[16] Creswell J. W (2012). Qualitative Inquiry and Research Design: Choosing Among Five Approaches. SAGE Publications.

[17] Miles MB, Huberman AM, Saldaña J Qualitative data analysis : a methods sourcebook. 2014 381.

[18] Rossiter K, Gray J, Kontos P, Keightle M, Colantino A, Gilbert J From Page to Stage: Dramaturgy and the Art of Interdisciplinary Translation. J Health Psychol. 2008;13(2):277-286. 18375632 10.1177/1359105307086707

[19] Abelson J, PPEET Research-Practice Collaborative Public and Patient Engagement Tool (PPEET). McMaster University; 2015.

[20] Hossain R, Moore M, Ramsay N, Milo M Healthcare First: Improving access to healthcare for the homeless and vulnerably housed in Niagara. [Internet]. 2017 Available at: http://homelesshub.ca/sites/default/files/HEART-Healthcare-First-2nd-Ed.pdf [Accessed November 20, 2017].

[21] Vyas A., Rodrigues V. C., Ayres R., Myles P. R., Hothersall E. J., & Thomas H Public health matters: Innovative approaches for engaging medical students. Medical Teacher. 2017;39(4):402-8. doi:10.1080/0142159x.2017.1294753 28379091

